# Clinical Features, Diagnosis, and Treatment of Eosinophilic Sialodochitis: A Systematic Review of the Literature

**DOI:** 10.1007/s12016-026-09160-8

**Published:** 2026-04-24

**Authors:** Alvaro Sánchez Barrueco, William Aragonés Sanzen-Baker, Marta Santiago Horcajada, Pilar Benavent Marín, Gonzalo Díaz Tapia, Jessica Mireya Santillán Coello, Virginia Ruiz San José, Christian Calvo-Henríquez, Carlos Cenjor Español, José Miguel Villacampa Aubá

**Affiliations:** 1 Facultad de Ciencias Biomédicas y de la Salud, Universidad Alfonso X el Sabio (UAX), Avenida de la Universidad 1, 28691 Villanueva de la Cañada, Madrid, Spain; 2 Salivary Pathology and Sialendoscopy Unit. ENT and Cervicofacial Surgery Department, Fundación Jiménez Díaz University Hospital, Av. de los Reyes Católicos, 2, Madrid, 28040 Spain; 3 Salivary Pathology and Sialendoscopy Unit. ENT and Cervicofacial Surgery Department, Villalba General University Hospital, Madrid, Spain; 4 Service of Otolaryngology, Hospital Complex of Santiago de Compostela, Santiago de Compostela, Spain; 5 Medicine Faculty, Universidad Autónoma de Madrid, Madrid, Spain

**Keywords:** Allergy, Eosinophilic, IgE, Mucous plug, Sialodochitis

## Abstract

Eosinophilic sialodochitis is an under-recognized obstructive inflammatory disorder of the major salivary ducts that has been described under heterogeneous terms, including allergic parotitis, fibrinous sialodochitis, and Kussmaul disease. We systematically reviewed the literature to summarize nomenclature, clinical presentation, diagnostic assessment, treatment, and consistency with proposed diagnostic criteria. We searched six bibliographic databases from inception to December 2025, complemented by Google Scholar and citation tracking. Studies describing eosinophilic sialodochitis or related entities were included irrespective of language. Data were synthesized descriptively because of marked heterogeneity. Seventy-nine studies involving 387 patients were included, comprising 64 full-text reports and 15 conference abstracts or posters. Most publications were case reports or case series. Recurrent parotid and/or submandibular swelling, eosinophil-rich salivary mucus plugs, ductal dilatation or stenosis, and frequent atopic comorbidity were the most characteristic findings. Eosinophils were identified in salivary mucus in 53 of 58 studies reporting local samples. Full compliance with published diagnostic criteria varied markedly, from 45 of 79 studies using the Baer framework to 2 of 79 using the Carey framework, while no study fully satisfied the Zhao framework. Treatment was usually multimodal and included antihistamines, corticosteroids, intraductal irrigation, sialendoscopy-assisted procedures, and, in selected refractory cases, biologic agents targeting type 2 inflammation. The available evidence supports eosinophilic sialodochitis as a distinct but incompletely standardized clinicopathological entity. Interpretation is limited by heterogeneity, incomplete reporting, and low-certainty evidence. Standardized definitions and minimum reporting items are needed to improve diagnostic certainty and comparability.

**Supplementary Information:**

The online version contains supplementary material available at 10.1007/s12016-026-09160-8.

## Introduction

In 1879, Kussmaul described what is considered the first historical report of fibrinous sialodochitis, a condition characterized by recurrent obstructive salivary swelling associated with ductal inflammatory material and microscopic findings consistent with eosinophilic activity.

Contemporary literature has progressively redefined the pathophysiological framework of these conditions, confirming obstruction by eosinophil-rich mucous plugs in a context frequently associated with atopy, hypersensitivity, and/or immunoallergic dysfunction [1–3]. This relationship is consistent with models of eosinophilic inflammatory disease mediated by mucus, described in other sites of the aerodigestive tract, where highly viscous mucus, eosinophilic activity, and findings such as Charcot–Leyden crystals or eosinophil extracellular traps (EETs) [4–7] contribute to both obstruction and tissue damage [8].

At the same time, one of the main challenges in interpreting and synthesizing the available evidence is terminological heterogeneity. Multiple terms have been used to refer, a priori, to the same nosological entity, including fibrinous sialodochitis or Kussmaul disease [1, 9–11], eosinophilic sialodochitis [1, 2, 5, 12] and eosinophilic sialadenitis [13, 14]; as well as allergic parotitis [15, 16] among others. This semantic dispersion affects the sensitivity of bibliographic searches, introduces classification bias, hinders comparison across case series, and favors the assignment of clinically similar conditions to different diagnostic labels.

In this context, several authors have proposed classification criteria aimed at defining the disease and avoiding the terminological dispersion. Accordingly, Baer et al. proposed a redefinition that shifts the focus from the fibrinous component toward a ductal eosinophilic phenotype, advocating the term *eosinophilic sialodochitis *[1]. Subsequently, Carey et al. refined this framework by proposing stricter criteria intended to improve diagnostic reproducibility and reduce the inclusion of clinically similar but etiologically distinct conditions [2]. Along a different line, Zhao et al. introduced the concept of *allergy-related sialodochitis* (ARS), integrating the phenomenon into a framework of allergic reactivity involving the major salivary glands and ductal changes [3]. The relevance of accurately characterizing these conditions is not limited to their acute semiology, as there is already evidence confirming that eosinophilic inflammation may be associated with tissue remodeling and fibrosis, potentially contributing to persistent glandular damage [17, 18].

Moreover, from a disease-burden perspective, it is important to emphasize that chronic obstructive/inflammatory salivary gland disorders may have a clinically relevant impact on symptoms and quality of life [14]. This further supports the interest in studying specific phenotypes and their evolution [14, 19, 20]. In this regard, a clearer delineation of the eosinophilic spectrum is a necessary step toward comparing outcomes, proposing diagnostic algorithms, and evaluating different treatment approaches.

For all these reasons, a systematic review is needed to organize the different terms and their conceptual equivalence, compare the proposed diagnostic criteria, synthesize clinical characteristics and findings (laboratory, histopathological, and complementary tests), treatments and outcomes, and identify evidence gaps and standardization needs, with the aim of moving toward more consistent and comparable definitions for research and clinical practice.

## Materials and Methods

A systematic review of the literature was conducted to address a structured research question based on the PICO framework (Population, Intervention, Comparator, and Outcomes). The review was designed in accordance with the PRISMA 2020 (Preferred Reporting Items for Systematic Reviews and Meta-Analyses) guidelines [21], with the aim of systematically evaluating eosinophilic sialodochitis (ES) and related entities. Specifically, we sought to describe the available clinical evidence, integrate the terminology used in the literature, review the proposed diagnostic criteria, and summarize reported therapeutic strategies and outcomes, including advanced medical treatments when documented. The study protocol was registered in PROSPERO (CRD420251251206).

Given the clinical and highly specialized nature of the condition under evaluation, observational studies were also included, encompassing case reports and case series. A meta-analysis was not performed due to the marked heterogeneity among the included studies in terms of design, diagnostic criteria applied, clinical heterogeneity of patients, sample size, and reported outcomes, as well as the frequent absence of comparator groups.

### PICO Question

The Population (P) included human patients (adult or pediatric) presenting with episodes of major salivary gland swelling and/or ductal involvement, with suspected or confirmed ES or equivalent/related entities.

The Intervention/Exposure (I/E) was defined as the presence of a salivary duct eosinophilic or allergic phenotype supported by compatible clinical, cytological, histopathological, and/or laboratory findings. Therapeutic interventions, when reported, were evaluated as outcomes rather than as part of the exposure definition.

The Comparator (C) encompassed any comparison framework reported in the original study, including alternative causes of salivary gland disease, non-eosinophilic phenotypes, or control groups when available. For descriptive non-analytical studies, such as case reports and case series, the absence of a comparator was not considered grounds for exclusion.

The Outcomes (O) included: terminology used, diagnostic criteria applied, clinical characteristics, diagnostic work-up (laboratory findings, cytology/biopsy, imaging/endoscopy), therapeutic approaches, clinical response/recurrence, and reported outcomes.

### Databases and Search Criteria

The literature search was conducted from database inception to 18 December 2025 in PubMed, Embase, Web of Science, Scopus, LILACS, and The Cochrane Library, without restriction by year of publication. A supplementary search was performed in Google Scholar to identify potentially non-indexed literature, and backward citation tracking of included studies and relevant publications was also conducted. Detailed search strategies are provided in Supplementary Material 1.

All references retrieved from the databases were imported into Rayyan (www.rayyan.ai) for management, screening, and tagging. Potential overlap between potentially related reports was assessed manually by cross-checking title, authorship, institution, recruitment period, and key clinical characteristics; no overlapping cohorts eligible for inclusion were identified in the final dataset. Two reviewers independently performed title/abstract screening and full-text assessment. Discrepancies were resolved through discussion until consensus was reached.

### Inclusion and Exclusion Criteria

Inclusion criteria comprised human studies providing primary data relevant to the review question, including case reports, case series, cohort studies, cross-sectional studies, and histopathological and/or radiological studies describing compatible cases with extractable data. Articles proposing diagnostic criteria or classifications were also eligible if they were based on real cases or presented an explicit and reproducible rationale. Conference abstracts/posters were included when they addressed the review question and provided sufficient clinical or diagnostic information for data extraction.

Exclusion criteria comprised in vitro studies, animal studies, studies not addressing the review question, and studies primarily focused on alternative diagnoses. These included benign or malignant salivary gland tumours, sialolithiasis, chronic non-eosinophilic obstructive sialadenitis, IgG4-related disease (including Mikulicz disease), Kimura disease, Sjögren’s syndrome, eosinophilic granulomatosis with polyangiitis (EGPA), histiocytosis or eosinophilic granuloma (including Langerhans and non-Langerhans cell histiocytoses such as Erdheim–Chester disease and Rosai–Dorfman disease), and subacute necrotizing sialadenitis. Because salivary plug extrusion alone may occur in obstructive salivary disorders unrelated to eosinophilic disease, studies describing plug extrusion without sufficient objective evidence of an eosinophilic or allergic phenotype were considered ineligible.

To avoid unnecessary exclusion of potentially relevant non-English studies, full-text articles published in languages other than English were translated using Google Translate and ChatGPT 5.2. The translations obtained from both tools were systematically compared for semantic equivalence, with particular attention to eligibility-relevant content, study design, interventions, and outcome data. When the two versions showed no material differences in interpretation, the translation was considered sufficiently robust for study selection and data extraction. This dual-tool approach was adopted to improve translation reliability while minimizing language bias.

### Data Extraction

For each included study, the following information was collected: bibliographic data, study design, patient and gland characteristics, terminology used, clinical presentation, duration of symptoms, laboratory findings, salivary cytology, or biopsy of the affected gland, as well as information on imaging or endoscopic evaluation, differential diagnoses considered, treatments administered, and reported outcomes.

Regarding laboratory parameters, peripheral eosinophilia was considered elevated when the absolute eosinophil count exceeded 0.5 × 10^9/L (≥ 500 cells/µL) [22]. Total IgE was considered elevated when it exceeded the upper limit of normal for adults (commonly > 100 kU/L or IU/mL) [23].

Ductal dilatation, stenosis, and sialectasis were extracted according to the terminology used in the original reports and were not retrospectively reclassified across older imaging modalities and more recent sialendoscopy-based descriptions.

Quantitative summaries were descriptive only and were calculated directly from the data reported in each study; no formal pooling or meta-analysis was performed. Weighted mean and weighted median age were calculated descriptively using the number of patients with available age data in each study as weights, and only studies with extractable age information were included in these summaries. To improve transparency regarding diagnostic certainty and potential misclassification, we added a study-level supplementary table (Supplementary Material 6) summarizing objective local eosinophilic evidence and overall diagnostic certainty for each included study.

## Methodological Quality Assessment

To evaluate the methodological quality of the included studies, critical appraisal tools were applied according to the design of each publication. All studies were assessed using the Joanna Briggs Institute (JBI) Critical Appraisal Checklists: for Case Reports (8 items), for Case Series (10 items), for Cohort Studies (11 items), and for Cross-Sectional Studies (8 items).

For descriptive interpretation, we applied a categorization based on the proportion of applicable JBI items rated as “Yes” (high, ≥ 75%; moderate, 50%–74%; low, < 50%). These categories were used only for descriptive comparison across study designs and do not represent an official JBI grading system.

### Assessment of Proposed Diagnostic Criteria

A standardized evaluation was conducted to assess compliance with the three diagnostic criteria sets proposed to date: Baer et al. [1], Carey et al. [2], and Zhao et al. [3]. The criteria are described in Table 1.

**Table 1:** Proposed diagnostic criteria for eosinophilic sialodochitis (ES) and allergy-related sialodochitis (ARS)

Author	Baer et al. (2017)[1]	Carey et al., 2022[2]	Zhao et al., 2021[3]
Disease designation	Eosinophilic sialodochitis	Eosinophilic sialodochitis	Allergy-related sialodochitis
Numbered criteria	B1. Recurrent/paroxysmal swelling of major salivary glands.	C1. Eosinophils in aspirated ductal contents.	Z1. Recurrent swelling of major salivary glands ≥ 3 months.
	B2. Intraductal mucous plugs with numerous eosinophils.	C2. Intermittent swelling of ≥ 1 major salivary gland.	Z2. ≥2 major salivary glands involved.
	B3. Peripheral eosinophilia and elevated IgE (operationalized as: AEC ≥ 500/mm³ or ≥ 5%; IgE > 111 kU/L).	C3. ≥1 additional symptom: glandular pruritus, or pain, or “string-like” mucus (plugs)	Z3. Allergic/atopic comorbidity.
	B4. Associated atopic disease.	C4. Exclusion of other causes of salivary gland swelling with eosinophils.	Z4. Ductal ectasia and/or stenosis (main duct and/or branches).
	B5. Ductal dilation (± focal stenosis).		Z5. Elevated peripheral eosinophils and/or elevated total IgE (in study: PBE > 0.52 × 10^9/L or > 8%; IgE > 100 IU/mL).
	B6. Histology: eosinophil/lymphocyte-rich periductal inflammation + fibrosis + reactive ductal epithelium		Z6. Intraductal mucous plugs with eosinophilic infiltration (plug cytology).
	B7. Exclusion: does not meet criteria for IgG4-related disease		Z7. Histology: eosinophil-rich inflammation around large ducts (periductal). Z8. Exclusion: IgG4-related disease and Kimura disease (and other relevant differentials).
Diagnostic rule	Mandatory:• B1 + B2 or• B1 + B6 + B7.	Mandatory: C1 + C2 + C3 + C4	Mandatory: Z1 + Z2 + Z3 + Z4.Suspicion/supportive: Z5.Verification/confirmation: Z6 or Z7.Additionally: apply Z8 (exclusions).

For each set of criteria, one of the following outcomes was assigned: YES (if the case(s) fulfilled the criteria), NO (if the case or all cases did not fulfill the criteria), or PARTIAL. Each required component of the criteria was assessed only if it was explicitly described; items that were not reported or were ambiguously described were coded as unclear, and fulfillment was not assumed.

The final classification (YES/NO/PARTIAL) was assigned on a case-by-case basis within each study. In case series, the study was labeled as PARTIAL when both cases fulfilling and cases not fulfilling the criteria coexisted within the same series.

## Results

The literature search identified a total of 7,410 records (6,964 retrieved from databases and 446 from other sources). After removal of duplicates, 3,625 references were screened in Phase 1 (title and abstract screening). Records not meeting the inclusion criteria were subsequently excluded.

**Fig. 1:**
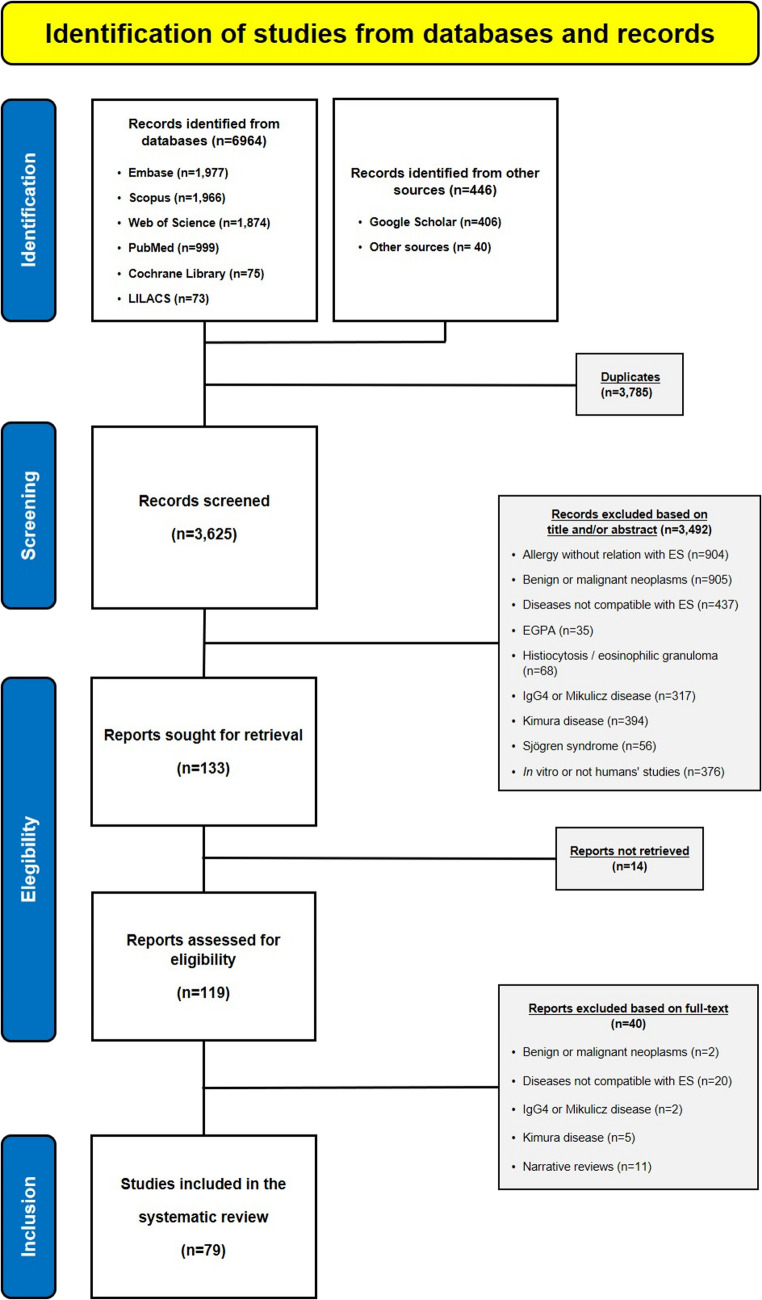
PRISMA 2020 flow diagram showing study identification, screening, eligibility assessment, and final inclusion, with itemized reasons for exclusion at the full-text stage

A total of 133 full-text reports were sought for retrieval, of which 14 could not be obtained. In Phase 2 (full-text assessment), 119 articles were evaluated, and 40 were excluded for the reasons detailed in the PRISMA flow diagram (Fig. 1).

Ultimately, 79 studies met the inclusion criteria and were incorporated into the qualitative synthesis of this systematic review. Unless otherwise specified, all frequencies presented in the Results refer to the number of studies reporting a given feature and should not be interpreted as patient-level prevalence estimates.

### General Characteristics of the Included Studies

The predominant language of publication was English (52; 65.82%), followed by Japanese (22; 27.85%). The remaining languages were represented by one article each (German, Italian, French, Chinese, and Russian).

The publication date range spanned from 1879 to 2025, with a greater concentration of publications in more recent years: before 2000 (*n* = 21; 26.58%), 2000–2020 (*n* = 28; 35.44%), and after 2020 (*n* = 30; 37.97%).

The included evidence was predominantly descriptive in nature, consisting of 64 full-text articles [1–7, 9–12, 14–16, 24–72] and 15 conference abstracts/posters [13, 18, 73–85] (Table 2). This distinction should be considered when interpreting aggregated findings.

**Table 2:** Distribution by study design and publication format among the studies included in the review

Study design	Article	Abstract/Poster	Total *n* (%)
Case report	38	13	51 (62.67%)
Case series	22	1	23 (30.67%)
Cohort study	3	0	3 (4%)
Cross-sectional study	1	1	2 (2.67%)
Total	**64**	**15**	**79 (100%)**

Data are expressed as n (%) relative to the total number of included studies. Percentages refer to studies, not patients

Across all included studies, 387 patients were reported. Despite this cumulative sample size, the evidence base remained overwhelmingly descriptive, with most publications being single-patient reports (*n* = 71; 89.87%). The largest sample sizes were reported by Zhao et al. (96 patients and 217 glands) [3] and Carey et al.. (37 patients, without specification of the number of affected glands) [2].

#### Methodological Quality

**Fig. 2:**
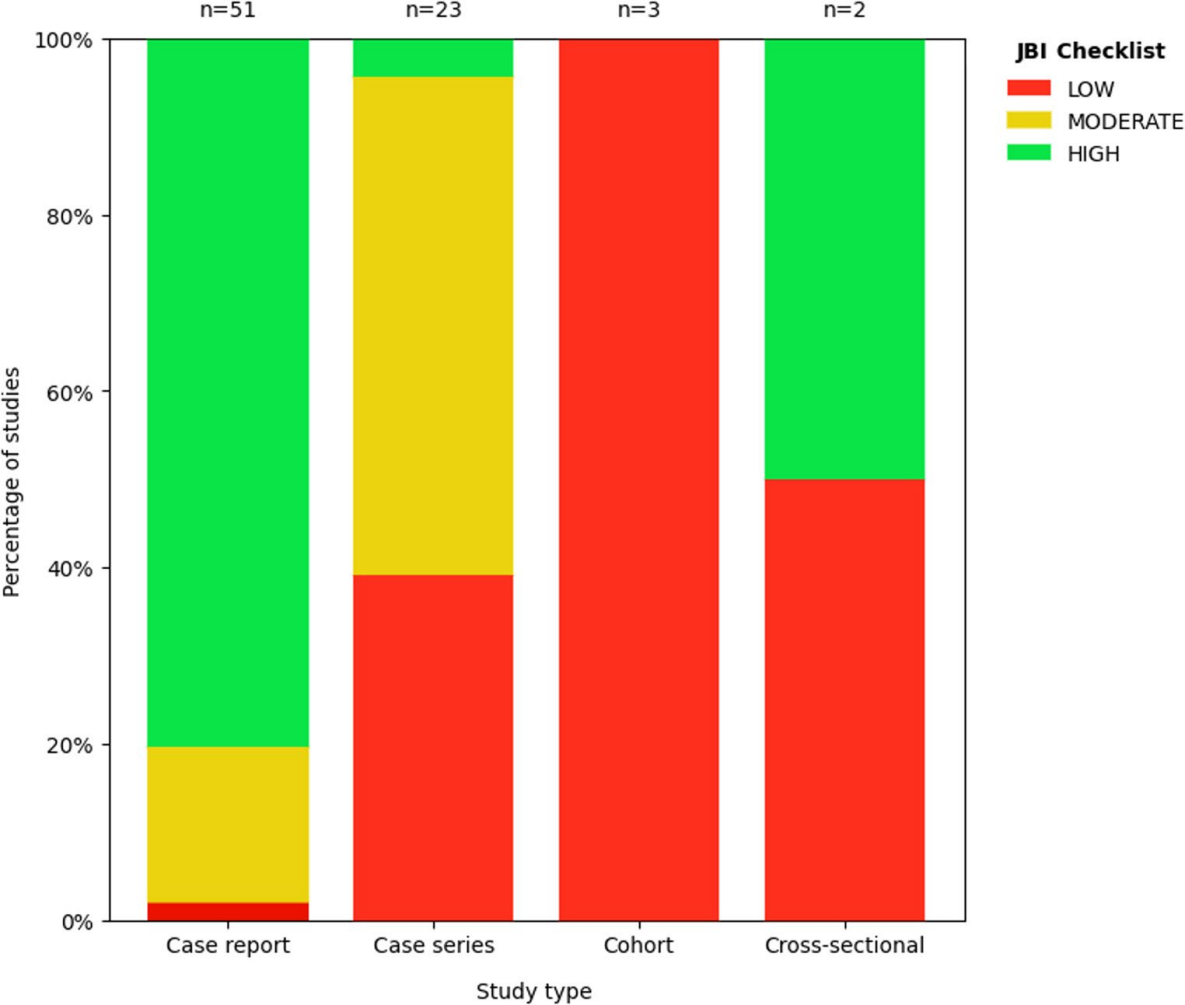
Distribution of methodological quality according to JBI by study design. Stacked bar chart showing the proportion of studies classified as low, moderate, and high quality within each study design

By study type, case reports predominantly demonstrated high methodological quality (41/51; 80.39%). Among case series, moderate quality was most frequent (13/23; 56.52%), followed by low quality (9/23; 39.13%). Cohort studies were classified as low quality in 100% of cases, while cross-sectional studies showed an equal distribution between low and high quality (Fig. 2). This low-quality rating in cohort studies was mainly driven by recurrent limitations in domains related to group comparability, identification and management of confounding, and completeness or clarity of follow-up.

All detailed quality assessment data are provided in Supplementary Material 2.

### Nomenclature Used

If a single study could employ more than one term to refer to the disease, the most frequently used terms were *fibrinous sialodochitis* (38/79; 48.10%) and *Kussmaul disease* (34/79; 43.04%). *Eosinophilic sialodochitis* was used in 27/79 studies (34.18%), whereas *eosinophilic sialadenitis/parotitis* appeared in 4/79 (5.06%).

The grouped terminology *allergic sialadenitis/parotitis* was identified in 14/79 studies (17.72%), and *allergy-related sialodochitis* (ARS), a term coined by Zhao et al. [3], was used in 2/79 (2.53%). Over time, an evolutionary trend can be observed, with *eosinophilic sialodochitis* emerging as the most used term in more recent studies.

All detailed information is provided in Supplementary Material 3.

### Demographics, Clinical Presentation, Atopy, and Gland Involvement

The weighted mean age was 43.17 years (*n* = 206; 53.23%), and the weighted median age was 48 years (*n* = 272; 70.28%). Pediatric involvement (< 18 years) was uncommon, without a specific age predominance, with some exclusively pediatric series [39, 45, 50, 59, 77] and others including mixed populations [56, 58, 69, 71].

The overall age range was broad, from 0.75 to 80.0 years. A clear female predominance was observed (243 women vs. 110 men; female-to-male ratio 2.21:1).

The most common symptoms, duration of symptoms, and associated allergic conditions are described in Table 3.

**Table 3:** Frequency of symptoms, potential triggers, duration, and atopic comorbidities reported in the included studies

Variable	%	*n*/*N*
Symptoms		
Recurrent swelling of salivary glands	82.28%	65/79
Mucous plug (mucofibrinous, fibrinous, filamentous “string-like,” or gelatinous “jelly-like”)	59.49%	47/79
Glandular pain or tenderness	49.37%	39/79
Glandular pruritus	17.72%	14/79
Symptom triggers		
Triggered by food intake	22.78%	18/79
Not triggered by food intake	12.66%	10/79
Duration of symptoms reported (median reported duration = 6 years)	64.56%	51/79
Atopic comorbidity		
Allergic rhinitis/allergic rhinoconjunctivitis	50.63%	40/79
Asthma	37.97%	30/79
Urticaria	18.99%	15/79
Atopic dermatitis/eczema	17.72%	14/79
Food allergy	10.13%	8/79
Angioedema	5.06%	4/79
Chronic rhinosinusitis with nasal polyps	3.80%	3/79
Eosinophilic esophagitis	2.53%	2/79

Data are expressed as n/N (%) relative to the total number of included studies. Percentages refer to studies, not patients, unless otherwise specified. All detailed information is provided in Supplementary Material 3

Across the included studies, a cumulative total of 252 parotid glands and 154 submandibular glands was reported among 387 patients. In 12 studies, the specific number or type of affected glands was not reported [6, 7, 13, 55, 69–73, 77, 78, 81].

**Fig. 3:**
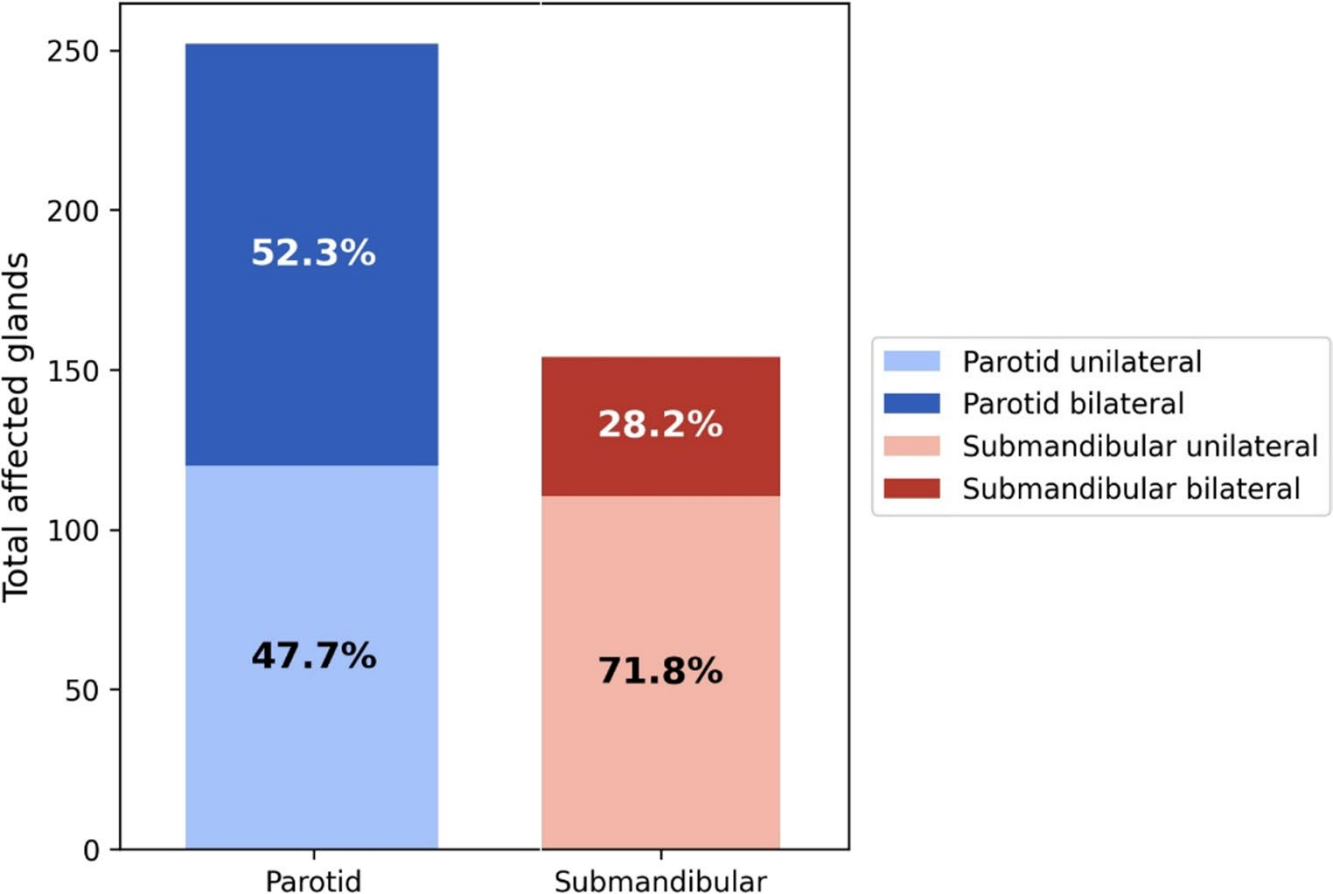
Cumulative affected glands and laterality. Counts of parotid and submandibular involvement are shown at the gland level, whereas laterality and mixed-gland involvement are summarized from patient counts aggregated across studies. These descriptive data should not be interpreted as patient-level or population-level prevalence estimates

Among studies reporting these variables, bilateral involvement (of either the parotid or submandibular glands) and mixed involvement (both parotid and submandibular glands) were summarized descriptively from the reported patient counts (Fig. 3). Mixed gland involvement was reported in 21 studies, with a weighted mean of 30.4%. In rare instances, involvement of the sublingual gland was also described [1, 57]. Cumulative parotid andsubmandibular involvement were summarized at the gland level, whereas laterality and mixed-gland involvement were derived from reported patient counts aggregated across studies.

All detailed information is provided in Supplementary Material 4.

### Diagnostic Work-Up

The diagnostic evaluations most frequently reported included laboratory testing, cytology or histology of salivary mucus, histology of the salivary gland, and imaging studies **(**Tables 4 and 5**).**

**Table 4:** Laboratory, mucus cytology/histology, and tissue biopsy findings

Reported complementary investigations	%	*n*/*N*
Laboratory testing		
Peripheral eosinophils	64.56%	51/79
* Elevated*	72.54%	37/51
Total IgE	54.43%	43/79
* Elevated*	83.72%	36/43
Cytology/Histology of salivary mucus	73.42%	58/79
Eosinophils +	91.38%	53/58
Charcot-Leyden crystals	6.9%	4/58
Tissue biopsy	30.38%	24/79
Tissue eosinophilia	50%	12/24

Data are expressed as n/N (%) relative to the total number of studies unless otherwise specified. For subitems such as “Elevated” or “Eosinophils present”, the denominator corresponds to studies reporting the relevant test

**Table 5:** Imaging, sialendoscopy, and structural ductal findings

Reported complementary investigations	%	*n*/*N*
Imaging studies	87.34%	69/79
Sialography	55.07%	38/69
CT	33.33%	23/69
Ultrasound	24.64%	17/69
Scintigraphy	21.74%	15/69
Plain radiography	18.84%	13/69
MRI	15.94%	11/69
Sialendoscopy	7.25%	5/69
MR Sialography	2.9%	2/69
Imaging findings		
Ductal dilatation	53.62%	37/69
Stenosis	20.29%	14/69
Sialectasis	7.25%	5/69

Data are expressed as n/N (%) relative to the total number of studies unless otherwise specified

Among studies specifying laboratory values, peripheral eosinophilia was elevated in 37/51 (72.54%), and total IgE was elevated in 36/43 (83.72%).

Cytological or histological examination of salivary mucus revealed the presence of eosinophils in 91.38% of cases (53/58), while only four studies reported the presence of Charcot–Leyden crystals [5, 24, 50, 86]. In contrast, tissue biopsy demonstrated eosinophilic infiltration in 50% of studies (12/24). Notably, five studies specifically described biopsy sampling from the terminal salivary duct [4, 29, 53, 71, 72].

Sialendoscopy was reported as a diagnostic or confirmatory procedure in 5/79 studies, all of which were published from 2022 onward (6.32%) [2, 14, 26, 49, 54]. This pattern likely reflects a temporal shift in diagnostic practice and reporting rather than a criterion consistently applied across the earlier literature.

The most frequently reported imaging findings were ductal dilatation (37/79; 46.84%), stenosis (14/79; 17.72%), and sialectasis (5/79; 6.33%). Only one study reported intraoral stone extraction [60].

All detailed information is provided in Supplementary Material 5. A study-level descriptive stratification according to the presence or absence of objective local eosinophilic evidence, together with overall diagnostic certainty, is provided in Supplementary Material 6.

#### Compliance with Diagnostic Criteria

Application of the diagnostic criteria revealed marked heterogeneity depending on the system used. In retrospective reports, lack of fulfillment should be interpreted cautiously, as non-fulfillment may reflect incomplete or ambiguously reported information rather than true absence of the required feature.

Using the criteria proposed by Baer et al. [1], more than half of the studies achieved full compliance (45/79; 56.96%), and 13.92% demonstrated partial compliance. The criteria most frequently not fulfilled were the reporting of intraductal eosinophil-rich mucous plugs (B2), exclusion of other diseases such as IgG4-related disease (B7), and the absence of compatible histological findings (B6).

In contrast, full compliance with the criteria proposed by Carey et al. [2] was exceptional. Only 2/79 studies (2.53%) achieved complete fulfillment, and 7/79 (8.86%) achieved partial fulfillment. Most studies were unable to exclude other causes of salivary gland swelling with eosinophilic infiltration (C4).

The situation was even more restrictive for the criteria proposed by Zhao et al. [3], as no studies achieved full compliance, and only 8.86% demonstrated partial fulfillment. Similar to the Carey criteria, most studies failed to adequately exclude other diseases (Z8).

All detailed information is provided in Supplementary Material 7.

#### Treatment, Follow-Up, and Outcome Assessment

Treatment approaches were highly heterogeneous and, in many cases, involved different combinations of therapeutic measures, making it difficult to assess overall effectiveness. In general, the predominant therapeutic pattern was stepwise, combining medical treatment for symptomatic control and, in persistent or recurrent cases, additional ductal interventions.

Regarding medical therapy, the most common approach focused on controlling type 2 inflammation and symptoms using antiallergic medications: antihistamines or other antiallergic agents (48/79; 60.8%) and systemic corticosteroids (24/79; 30.4%). Less frequently reported treatments included antibiotics (10/79; 12.7%), montelukast or other leukotriene receptor antagonists (8/79; 10.1%), and mucolytics (7/79; 8.9%).

Conservative ductal management through saline irrigation or lavage was frequently described (31/79; 39.2%), sometimes combined with intraductal corticosteroids (5/79; 6.3%) [1–3, 49, 50].

The use of biologic agents was reported in 8/79 studies (10.1%), primarily targeting type 2 inflammatory pathways: omalizumab (anti-IgE) [25, 72], dupilumab (anti-IL-4Rα) [26, 77], mepolizumab (anti-IL-5) [2, 81], and benralizumab (anti-IL-5Rα) [2, 25, 49, 55]. In only a few cases were validated outcome measures employed [55, 81], as in most studies the indication for treatment and assessment of response were reported in a non-standardized manner. Nevertheless, symptom improvement following treatment was reported in most cases.

Regarding interventional and surgical management, ductal dilation without sialendoscopy was reported in 10/79 studies (12.7%) [3, 13, 31, 42, 46, 50, 51, 71, 82, 86], while sialendoscopy-assisted ductal dilation was described in 14/79 studies (17.7%)[1–3, 14, 26, 46, 49–51, 53, 70, 71, 81, 82]. In these cases, sialendoscopy often fulfilled both diagnostic and therapeutic roles, particularly through the evacuation of mucous plugs. Ductal splint or stent placement was reported in 5/79 studies (6.3%) [2, 26, 50, 53, 81]. More invasive procedures were reported less frequently, including gland excision (13/79; 16.5%) and salivary duct resection (4/79; 5.1%).

Reporting of follow-up duration was limited or not extractable in 40/79 studies (50.6%). Among those specifying follow-up time, the median duration was 12 months.

Disease course and treatment outcomes were highly variable, and the use of standardized outcome or quality-of-life measures was infrequent (5/79; 6.33%) [2, 3, 14, 72, 81]. Due to the absence of validated quality of life assessment tools in most studies, outcomes were categorized in aggregate as follows: improvement (38/79; 48.1%), complete remission (15/79; 19%), partial remission (7/79; 8.9%), variable response (10/79; 12.7%), or no change (1/79; 1.3%). Recurrence or persistence was explicitly documented in a minority of studies (13/79; 16.5%), limiting analysis of medium- to long-term evolution.

All detailed information is provided in Supplementary Material 8.

## Discussion

The present systematic review confirms that eosinophilic sialodochitis (ES) and related entities remain a field characterized by marked terminological heterogeneity, methodological variability, and insufficiently standardized outcome reporting, all of which limit comparability and hinder robust conclusions. Accordingly, the aggregated frequencies reported in this review should be interpreted as descriptive signals rather than pooled estimates of prevalence, diagnostic performance, or treatment effect. Across the included literature, the most frequently used designations were *fibrinous sialodochitis*, *eosinophilic sialodochitis*, and *Kussmaul disease*, whereas more recent terms such as *allergy-related sialodochitis* (ARS) were used less often [3, 72]. This semantic variability is clinically relevant, as it hinders homogenization of case series despite the presence of a shared clinical phenotype characterized by recurrent salivary gland swelling, extrusion of mucoid plugs, and atopic comorbidity. Based on the available evidence, eosinophilic sialodochitis appears to be the most appropriate designation, as it reflects both the eosinophilic nature of the process and its predominantly duct-centered involvement, with secondary extension to the salivary gland parenchyma.

The evidence base is dominated by case reports, which, despite often showing good reporting quality, have limited inferential value and are susceptible to publication bias. Conference abstracts/posters and older historical reports further reduce evidentiary strength because they frequently provide less complete diagnostic, methodological, and outcome detail than peer-reviewed full-text studies. Together with selective reporting and non-standardized outcome assessment, these factors result in low overall certainty of the available evidence.

Across the included studies, the condition is characterized by recurrent swelling of the major salivary glands (predominantly parotid and sometimes bilateral), often triggered by food intake or mastication and partially relieved by ductal expression. This is frequently accompanied by the extrusion of viscous mucous plugs, described as filamentous (“string-like”) or gelatinous (“jelly-like”). When reported, the clinical course is typically prolonged (median duration of 6 years), and atopic comorbidity—particularly allergic rhinitis/rhinoconjunctivitis and/or asthma—is common. The diagnostic profile is supported by demonstration of eosinophils in ductal material (cytology/histology), suggestive obstructive findings on complementary studies, and a heterogeneous response to antiallergic/anti–type 2 therapies and intraductal measures or sialendoscopy.

The diagnostic cornerstone lies in the analysis of salivary mucus, either by cytology or histology, as well as glandular biopsy or biopsy of the major salivary duct [4, 29, 53, 71, 72]. A major source of potential misclassification in the literature is the reporting of salivary plug extrusion without objective eosinophilic or allergic evidence, which may reflect non-eosinophilic obstructive salivary disease rather than true eosinophilic sialodochitis. More recently, some authors have emphasized that thorough processing of mucoid plugs not only reveals abundant eosinophils but also ductal epithelial cells with eosinophilic infiltration and metaplastic changes. It has thus been postulated that activated eosinophils may contribute to plug formation and stabilization, potentially through EETosis, which could increase viscosity and promote aggregation of mucus and debris [4–7]. In addition to the mucoid plug itself, obstruction has been confirmed by radiologic imaging and, in recent years, by sialendoscopy [2, 14, 26, 49, 54]. More broadly, the diagnostic pathway has changed substantially over time: older reports relied mainly on clinical presentation and conventional imaging, whereas more recent studies increasingly incorporate mucus cytology, targeted ductal or gland biopsy, immunopathological characterization, MR-based ductal imaging, and sialendoscopy. This temporal shift must be considered when comparing historical and contemporary reports. These findings indicate that the disease is commonly associated with ductal dilation, stenosis, and sialectasis. However, the terminology used for these ductal abnormalities was not fully standardized across the historical literature, and some variability likely reflects differences in diagnostic modality and reporting conventions rather than strictly distinct pathoanatomical entities.

Regarding pathogenesis, more recent literature converges toward a type 2 (Th2) inflammatory framework, particularly in patients with allergic comorbidity or asthma [8, 26, 35, 72], although the evidence remains largely indirect and heterogeneous. Several studies provide biologically plausible signals, including tissue immunostaining for markers associated with eosinophils and mast cells such as IL-4, IL-13, CCR3, eotaxin, and tryptase [69, 72], IgE-independent mechanisms mediated by mast cell degranulation (histamine/tryptase) and eosinophilic mediators [6, 12], deposits of eosinophil major basic protein [4, 5], and elevated salivary IL-5 levels [35, 65].

Different diagnostic criteria have been developed, notably those proposed by Baer et al. [1], Carey et al.[2], and Zhao et al.[3]. All three converge on a common conceptual core: recurrent episodes of major salivary gland swelling with evidence of eosinophilic inflammation (eosinophil-rich plugs/mucus or biomarkers such as peripheral eosinophilia and/or elevated IgE), together with the need to exclude relevant alternative diagnoses, particularly IgG4-related disease. This distinction is particularly relevant in relation to clinical mimickers such as sialolithiasis and chronic non-eosinophilic obstructive sialadenitis, which may also present with recurrent swelling and obstructive symptoms. However, they differ substantially in how this core is operationalized into specific requirements. Baer et al. allow two diagnostic pathways (clinical features plus plugs, or clinical features plus histology plus exclusion of IgG4-related disease) [1], whereas Carey et al. place greater emphasis on cytology, requiring eosinophils in aspirated ductal content in addition to clinical symptoms and exclusion criteria [2]. Zhao et al. propose a stepwise scheme with mandatory criteria (duration ≥ 3 months, involvement of ≥ 2 glands, atopic comorbidity, and ductal findings) combined with supportive criteria (eosinophilia/elevated IgE) and confirmation by plug or histology [3]. This definitional heterogeneity likely contributes to variability in fulfillment rates across studies and increases the risk of misclassification in retrospective series or conference abstracts. Potential misclassification bias is particularly relevant in the older literature, especially in reports published before modern cytological, histopathological, and immunological assessment became routinely available. In such studies, diagnosis was often inferred from recurrent swelling and ductal obstruction alone, without objective eosinophilic confirmation, which limits comparability with more recent reports. From a practical perspective, the three frameworks may be interpreted schematically as follows. Baer et al. [1] provide the broadest and most inclusive diagnostic construct, potentially capturing a wider historical clinical spectrum. Carey et al. [2] offer the most operational and clinically reproducible framework for contemporary practice, as they rely on a relatively clear combination of clinical symptoms, direct ductal eosinophilic evidence, and exclusion of alternative diagnoses. Zhao et al. [3] provide the most structured phenotypic framework and may be particularly useful for stratification and research standardization. Because comparative diagnostic accuracy studies are lacking, these distinctions should be interpreted in conceptual and clinical terms rather than as formal estimates of sensitivity or specificity.

Treatment of ES is almost invariably multimodal, which makes objective assessment of therapeutic effectiveness challenging. The most commonly used medications were antihistamines and systemic corticosteroids, followed by leukotriene receptor antagonists and mucolytics. Intraductal irrigation was frequently performed, either alone or combined with corticosteroids [1–3, 49, 50]. Over time, sialendoscopy has increasingly been incorporated as both a therapeutic and diagnostic modality, enabling intraductal irrigation while also confirming the presence of mucous plugs and ductal stenosis [1–3, 14, 26, 46, 49–51, 53, 70, 71, 81, 82]. In refractory cases, gland excision or salivary duct resection was occasionally reported.

The incorporation of biologic agents has emerged as an area of growing interest, although the available evidence remains very limited (8/79 studies; 10.1%), primarily targeting type 2 inflammatory pathways (anti-IgE, anti-IL-4Rα, anti-IL-5, anti-IL-5Rα) [2, 25, 26, 49, 55, 77, 81, 83]. Across these reports, both indications and treatment responses were described in a non-standardized manner, typically in the context of asthma or other allergic comorbidities and refractoriness to conventional therapies. The available reports suggest a pragmatic stepwise pattern in clinical practice, usually starting with medical treatment and/or intraductal irrigation and, in selected cases, progressing to sialendoscopy or other interventions. However, this pattern should be interpreted as a descriptive observation from heterogeneous reports rather than as evidence-based therapeutic guidance. More aggressive interventions, including surgery or systemic rescue treatment, should remain individualized options for highly selected patients.

It is essential to consider that improvement attributed to biologic therapy may be confounded by concomitant treatments (by the clinical evolution of associated comorbidities, and by the relapsing and potentially fluctuating course of ES itself. Nevertheless, many reports describe favorable outcomes with biologic use. In 4/8 studies, marked symptomatic reduction was reported with dupilumab [26, 77], benralizumab [25], or mepolizumab [81]. In 2/8 studies, the response to benralizumab was partial or heterogeneous [49, 55]. Conversely, in 2/8 studies no salivary improvement was observed with mepolizumab or benralizumab [2], or salivary response was not specifically assessed in patients treated with omalizumab [72]. This heterogeneity is partly explained by the fact that biologic therapy in ES is usually driven by concomitant atopic indications (e.g., asthma or atopic dermatitis) rather than by a specific indication for ES itself. This circumstance complicates the definition of a homogeneous “index patient” and hinders standardization of both selection criteria and therapeutic response assessment.

Finally, a cross-cutting limitation is the scarce adoption of standardized outcome or quality-of-life measures. Such tools were used in only 5 studies [2, 3, 14, 81, 83], based on the COSS [87], COSQ [88, 89], MSSS [90], and G-SESS questionnaires [2]. Broader implementation of these instruments represents a potential opportunity to improve therapeutic outcome assessment and should be considered in future studies. Additionally, follow-up duration was limited; when reported, the median follow-up was 12 months.

## Conclusions

Eosinophilic sialodochitis appears to be an under-recognized type 2-associated obstructive salivary duct disorder characterized by recurrent gland swelling and, when documented, eosinophil-rich mucous plugs. Nevertheless, the current evidence base remains predominantly descriptive, heterogeneous, and largely limited to small observational reports with variable terminology and incomplete reporting, which frequently precludes reproducible application of proposed diagnostic criteria and requires cautious interpretation of available therapeutic data. Taken together, the literature supports a more structured diagnostic framework integrating allergic comorbidity, focused laboratory testing, salivary mucus analysis, and targeted imaging or ductal evaluation, while underscoring the need for unified terminology, explicit standardized diagnostic definitions, minimum reporting items, and prospective outcome studies with robust quality-of-life measures before definitive therapeutic recommendations can be established.

## Supplementary Information

Below is the link to the electronic supplementary material.

Supplementary Material 1 (DOCX 16.1 KB)

Supplementary Material 2 (XLSX 26.5 KB)

Supplementary Material 3 (XLSX 133 KB)

Supplementary Material 4 (XLSX 15.3 KB)

Supplementary Material 5 (XLSX 20.0 KB)

Supplementary Material 6 (XLSX 26.7 KB )

Supplementary Material 7 (XLSX 26.4 K)

Supplementary Material 8 (XLSX 21.6 KB)

## Data Availability

All extracted data are provided in Supplementary Materials.

## Abbreviations

ARS: Allergy-Related Sialodochitis
EETs: Eosinophil extracellular traps
ES: Eosinophilic sialodochitis or Eosinophilic sialadenitis
JBI: Joanna Briggs Institute
PRISMA: Preferred Reporting Items for Systematic Reviews and Meta-Analyses
