# Supplementary material for: Clinical Features, Diagnosis, and Treatment of Eosinophilic Sialodochitis: A Systematic Review of the Literature
Source: Clin Rev Allergy Immunol. 2026 Apr 24;69(1):36. doi: 10.1007/s12016-026-09160-8 (PMC13109154; doi:10.1007/s12016-026-09160-8)
Supplement: Supplementary file 1 — Supplementary Material 1 (DOCX 16.1 KB) [file 12016_2026_9160_MOESM1_ESM.docx]

**PubMed**

(("Salivary Glands"[Mesh] OR "Salivary Gland Diseases"[Mesh] OR "Salivary Ducts"[Mesh] OR "Sialadenitis"[Mesh] OR salivary gland*[tiab] OR salivary duct*[tiab] OR sialadenitis[tiab] OR sialodochitis[tiab] OR parotid[tiab] OR submandibular[tiab] OR submaxillary[tiab] OR sublingual[tiab]) AND ("Eosinophilia"[Mesh] OR "Eosinophils"[Mesh] OR eosinophil*[tiab] OR "eosinophilic granulomatosis with polyangiitis"[tiab] OR "Churg-Strauss"[tiab] OR EGPA[tiab] OR "hypereosinophilic syndrome"[tiab])) AND Humans[Mesh]

**Embase**

(('salivary gland'/exp OR 'salivary gland disease'/exp OR 'salivary duct'/exp OR 'sialadenitis'/exp OR 'salivary gland*':ti,ab OR 'salivary duct*':ti,ab OR sialadenitis:ti,ab OR sialodochitis:ti,ab OR parotid:ti,ab OR submandibular:ti,ab OR submaxillary:ti,ab OR sublingual:ti,ab) AND ('eosinophilia'/exp OR 'eosinophil'/exp OR eosinophil*:ti,ab OR 'eosinophilic granulomatosis with polyangiitis':ti,ab OR 'Churg-Strauss':ti,ab OR EGPA:ti,ab OR 'hypereosinophilic syndrome':ti,ab)) AND 'human'/de

**Web of Science**

TS=(("salivary gland*" OR "salivary duct*" OR sialadenitis OR sialodochitis OR parotid OR submandibular OR submaxillary OR sublingual) AND (eosinophil* OR "eosinophilic granulomatosis with polyangiitis" OR "Churg-Strauss" OR EGPA OR "hypereosinophilic syndrome"))

**Scopus**

TITLE-ABS-KEY(("salivary gland*" OR "salivary duct*" OR sialadenitis OR sialodochitis OR parotid OR submandibular OR submaxillary OR sublingual) AND (eosinophil* OR "eosinophilic granulomatosis with polyangiitis" OR "Churg-Strauss" OR EGPA OR "hypereosinophilic syndrome"))

**Cochrane Library**

(salivary gland* OR salivary duct* OR sialadenitis OR sialodochitis OR parotid OR submandibular OR submaxillary OR sublingual):ti,ab,kw AND (eosinophil* OR "eosinophilic granulomatosis with polyangiitis" OR "Churg-Strauss" OR EGPA OR "hypereosinophilic syndrome"):ti,ab,kw

**LILACS**

tw:("salivary gland*" OR "salivary duct*" OR sialadenitis OR sialodochitis OR parotid* OR submandibular* OR submaxillary* OR sublingual*) AND tw:(eosinophil* OR "eosinophilic granulomatosis with polyangiitis" OR "Churg-Strauss" OR EGPA OR "hypereosinophilic syndrome")

**Google Scholar**

1. "eosinophilic sialadenitis"
2. "eosinophilic sialodochitis"
3. ("sialodochitis fibrinosa" OR "Kussmaul disease") AND (parotid OR submandibular OR salivary)
4. (dupilumab OR mepolizumab OR benralizumab OR omalizumab) AND (parotid OR salivary) AND (eosinophil OR eosinophilic) AND (sialadenitis OR parotitis)
5. "allergic parotitis"
